# The Impact of Inulin and a Novel Synbiotic (Yeast *Saccharomyces cerevisiae* Strain 1026 and Inulin) on the Development and Functional State of the Gastrointestinal Canal of Calves

**DOI:** 10.1155/2021/8848441

**Published:** 2021-01-05

**Authors:** S. Jonova, A. Ilgaza, M. Zolovs

**Affiliations:** ^1^Latvia University of Life Sciences and Technologies, Faculty of Veterinary Medicine, Preclinical Institute, Helmana Iela 8, Jelgava, LV-3004, Latvia; ^2^Daugavpils University, Institute of Life Sciences and Technology, Department of Biosystematics, Parades Iela 1a, Daugavpils, LV-5401, Latvia

## Abstract

Successful management of the dairy industry is closely related to rearing healthy calves. The proper development of the gastrointestinal canal is crucial to reach this goal. One of the strategies to promote this development is the addition of feed additives to the diet. This research aimed to determine the impact of prebiotic inulin and a new, not commercially available synbiotic (mix of prebiotic inulin and probiotic *S. cerevisiae* strain 1026) on the development of the gastrointestinal canal of calves by comparing the weight of the stomach, its relative ratio to body weight and evaluating pH, and histological changes in different parts of the gastrointestinal canal and assess whether or not the addition of inulin to the yeast *S. cerevisiae* improves the abovementioned parameters. We used prebiotic inulin (6 g) and a synbiotic (prebiotic inulin 6 g and probiotic *Saccharomyces cerevisiae* strain 1026, 5 g). The 56-day long research was conducted with fifteen crossbreed calves (32 ± 6 days old) organized in the control group (CoG), the prebiotic group (PreG), and the synbiotic group (SynG). We determined pH, morphological parameters of different parts of the digestive canal, and morphometric parameters of the stomach. The addition of prebiotic inulin to calves' diet causes the increase of pH in rumen, abomasum, and intestines but when inulin was added to *S. cerevisiae*, pH decreased and was even lower than in the control group. Prebiotic inulin and its synbiotic with yeast *S. cerevisiae* positively impact the development of almost all morphological structures of rumen *saccus dorsalis*, rumen *saccus ventralis*, and intestine; moreover, calves from the synbiotic group showed better results in virtually all parameters. However, both inulin and synbiotic did not affect the weight and relative weight of different parts of the stomach. Tested synbiotic has the potential to promote the development of the rumen and other parts of the digestive canal of calves.

## 1. Introduction

Raising healthy calves in the dairy industry is essential as it directly impacts their development and future quality and quantity of produced milk. The proper development of the gastrointestinal canal is crucial to reach this goal. The rumen is an essential organ of the gastrointestinal canal that plays a major role in the growth and development of ruminants. At birth, the rumen is not fully developed, and it takes some time for it to start functionating at the level of adult ruminants. The specific changes happen in the rumen, including the development of rumen epithelium and the establishing of rumen microbiota [1]. The rumen only starts to grow at two to three weeks of age, and its growth will continue until about six months of age [2]. At birth, the weights of reticulorumen, omasum, and abomasum account for 38%, 13%, and 49% of the whole stomach weight, respectively. At 12 to 16 weeks of age, they reach 67%, 18%, and 15% of the stomach weight, respectively [1]. The abomasum is the only compartment of the whole stomach that is fully developed in newborn calves. Later on, the rumen begins to develop and starts to play a more important role in digestive processes. A rumen is well developed if the number of papillae and their size have greatly increased, so the total absorption surface is bigger and more nutrients can be absorbed [3]. The epithelium of rumen has many vital functions, and it plays a key role in rumen development, including absorption of nutrients, their transportation to other parts of the body, metabolism of short-chain fatty acids, and protection of rumen. The growth and proliferation of the rumen squamous epithelium contribute to the growth of papillae length and width and increase the thickness of the whole rumen wall [4]. The papillae length and width are the most important factors for the evaluation of rumen [5].

A lot of strategies describe how to promote the development of the morphological structure and metabolic function of the rumen and other parts of the gastrointestinal canal in calves and one of them is the addition of different feed additives to the diet such as probiotics and synbiotics. A wide variety of feed additives used for calves are available on the market. In 2008, the 6^th^ Meeting of the International Scientific Association of Probiotics and Prebiotics (ISAPP) defined “dietary prebiotics” as “a selectively fermented ingredient that results in specific changes in the composition and/or activity of the gastrointestinal microbiota, thus conferring benefit(s) upon host health” [6]. Probiotics are viable and beneficial microorganisms that help maintain microbial balance in the gastrointestinal canal and promote rumen development [7, 8]. For example, probiotic yeast *Saccharomyces cerevisiae* (*S. cerevisiae*) positively impacts ruminal microbiota by increasing rumen total bacteria, fungi, and protozoa [9] and improving ruminal morphology [10, 11]. Saccharomyces cerevisiae provides growth factors, including organic acids, B vitamins, and amino acids that stimulate microbial growth in the rumen, indirectly stabilizing ruminal pH [12]. Unlike probiotics, prebiotics act as a “food” for the target microbes with beneficial consequences for a host [13]. Prebiotics are resistant to gastric acidity, absorption, and hydrolysis by enzymes produced in the gastrointestinal canal [14]. The fermentation products of prebiotics by intestinal microflora also provide benefit by decreasing intestinal pH, which suppresses pathogenic bacteria [15, 16]. There are a lot of different types of prebiotics used in the livestock industry, such as fructooligosaccharides (FOS), galactooligosaccharides (GOS), mannan oligosaccharides (MOS), beta-glucans, inulin, and lactulose [17, 18]. Inulin is a polysaccharide consisting of fructose joined by a *β* 2,1 glycosidic bond containing small amounts of glucose [19]. Inulin belongs to a class of carbohydrates known as fructans. The primary sources of inulin are chicory (*Cichorium intybus* L.) and Jerusalem artichoke (*Helianthus tuberosus* L.) [20]. Inulin is not digested and absorbed in the small intestine but is selectively and quickly fermented by bacteria in lower parts of the gastrointestinal canal, stimulating proliferation of different *Lactobacillus*, mainly *Bifidobacterium*. Inulin has a beneficial effect on metabolism. It nourishes intestinal cells and lowers intestinal pH and stimulates the lengthening of intestinal villi as well as an increasing number of epithelial cells in individual villus [19]. Synbiotics are defined as “a mixture, comprising live microorganisms and substrate(s) selectively utilized by host microorganisms, that confers a health benefit on the host” [21]. The combination of a probiotic and a prebiotic can have a synergistic beneficial effect. The combination of two feed additives can improve the survival and the implantation of a direct-fed microbial in the gastrointestinal canal and stimulate the growth and activate the metabolism of a limited number of beneficial bacteria [22].

This research aimed to determine the impact of prebiotic inulin and a new, not commercially available synbiotic (mix of prebiotic inulin and probiotic *S. cerevisiae*) on the development of the gastrointestinal canal of calves by comparing the weight of the stomach and its relative ratio to body weight and evaluating pH and histological changes in different parts of the gastrointestinal canal, as well as assessing whether or not the addition of inulin to the yeast *S. cerevisiae* improves the abovementioned parameters.

## 2. Materials and Methods

### 2.1. Ethical Approval

All procedures performed in studies involving animals were in accordance with the ethical standards. Research Committee of the Faculty of Veterinary Medicine, Latvia University of Life Sciences and Technologies, approved this study (protocol no. 2017/2).

### 2.2. Study Area and Experimental Design

The study was completed from March till the end of April 2018 on a dairy farm which is located in Jaunlutrini Parish (latitude: N 56°49′42.2″, longitude: E 22°24′21.8″), Latvia. Fifteen clinically healthy randomly selected Holstein Friesian and Red Holstein (Bos *Taurus* L.) crossbreed calves of mean age of 32 ± 6 days and initial body weight of 72.1 ± 11.34 kg were used in the research. All calves were housed in groups in a partly closed space in a farm. After the birth, all calves received colostrum, and, for the next five days, calves received whole milk (3.5 l twice a day) and later the milk replacer in a dosage appropriate to the age and weight of the calves. Calves from four to eight weeks of age received eight litres of milk replacer per calf/day and a prestarter without restriction (around 0.5 kg per calf/day). From eight weeks of age, they received approximately 1.5 kg of barley flour per calf/day, six litres of milk replacer per calf/day. All the time, calves were given access to hay and water *ad libitum*. Calves were allocated into three groups: five calves in the control group (CoG) receiving a standard diet, five calves in the prebiotic group (PreG) that additionally received 12 g of flour of Jerusalem artichoke (*Helianthus tuberosus* L.) per head (50% of which or 6 g is prebiotic inulin) (produced in Latvia at the University of Latvia Institute of Microbiology and Biotechnology), and five calves in the experimental group (SynG) that additionally received synbiotic—12 g of flour of Jerusalem artichoke (*Helianthus tuberosus* L.) per head containing 6 g of prebiotic inulin and 5 g of probiotic *Saccharomyces cerevisiae* strain 1026 (Yea-Sacc®, Alltech Inc, USA). The prebiotic and probiotic were added to barley flour once a day in the morning. The duration of the experiment was eight weeks (56 days).

### 2.3. Sampling and Examination

**Figure 1:**
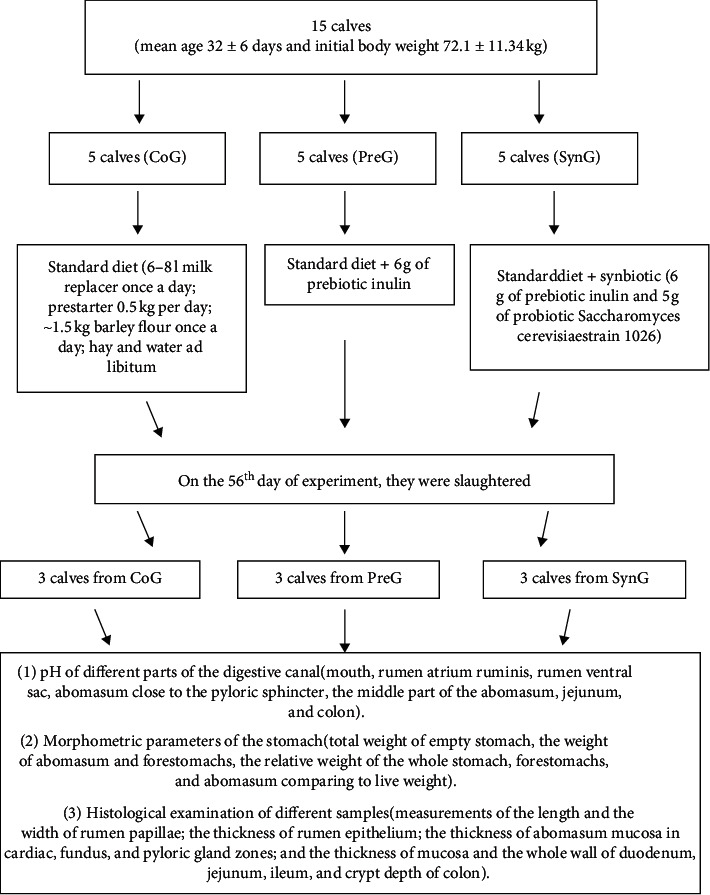
Full experimental design.

At the end of the experiment, three calves from each group were slaughtered at a certified slaughterhouse following all guidelines of humane slaughter. We measured the pH of different parts of the digestive canal (mouth, rumen *atrium ruminis*, rumen ventral sac, abomasum close to the pyloric sphincter, the middle part of the abomasum, jejunum, and colon) by using portable meter ProfiLine pH 3110, Xylem Inc. and determined morphometric parameters of the stomach (total weight of empty stomach, the weight of abomasum and forestomachs, the relative weight of the whole stomach, forestomachs, and abomasum comparing to live weight). The histological examination of different samples obtained from the gastrointestinal canal was performed. Measurements of the length and the width of rumen papillae; the thickness of rumen epithelium; the thickness of abomasum mucosa in cardiac, fundus, and pyloric gland zones; and the thickness of mucosa and the whole wall of duodenum, jejunum, ileum, and crypt depth of colon were performed by using microscope Leica DM 750, Leica Microsystems and software Leica Application Suite, and Leica Microsystems. Experimental design can be seen in Figure 1.

### 2.4. Statistical Analysis

The assumption of normal data distribution was assessed by Shapiro–Wilk's test and visual inspection of their histograms and normal Q-Q plots. The assumption of homogeneity of variances was tested by Levene's test. To determine whether there are any statistically significant differences between three or more independent groups, we used the Kruskal–Wallis H test with pairwise comparisons using Dunn's procedure [23] with a Bonferroni adjustment. To determine whether there are any statistically significant differences between the two groups, we used the Mann–Whitney *U* test or the independent samples *t*-test. The measure of the strength and direction of the association between two continuous or ordinal variables was evaluated by Spearman's rank-order correlation. Those tests were carried out using SPSS Statistics version 22 (IBM Corporation, Chicago, Illinois). All statistical analyses were performed at the significance level of *α* = 0.05.

## 3. Results

### 3.1. The pH in Different Parts of the Gastrointestinal Canal

One-way ANOVA showed that the pH of a mouth cavity significantly differs between treatment groups (*p* = 0.009). Tukey's post hoc test revealed that the pH of the mouth cavity in CoG (7.9 ± 0.25) was significantly lower than in the PreG (8.6 ± 0.35), and the pH in SynG (7.6 ± 0.21) was significantly lower than in Pre12. The pH of rumen *atrium ruminis* in CoG (7.1 ± 0.12) was significantly higher than in SynG (4.7 ± 0.15), and it was significantly higher in PreG (7.5 ± 0.85) than in SynG. The pH of rumen ventral sac in both CoG (7.1 ± 0.10) and PreG (7.5 ± 0.60) was significantly higher than in SynG (4.8 ± 0.30). The pH of the middle part of the abomasum in CoG (2.1 ± 0.25) was significantly lower than in PreG (3.8 ± 0.70), but, in SynG (2.0 ± 0.35), it was significantly lower than in PreG. The pH of the middle part of the colon in both CoG (6.0 ± 0.12) and PreG (6.5 ± 0.38) was significantly higher than in SynG (5.3 ± 0.15) (Table 1).

### 3.2. Morphometric Parameters of Stomach

In addition, there were no significant differences between the weight and relative weight of all forestomachs, abomasum, and whole stomach between both groups, *p* > 0.05 (Table 2).

The percentual volume of abomasum was 16.2% in CoG, 14.6% in PreG, and 19.8% in SynG, but the percentual volume of forestomachs was 83.8% in CoG, 85.4% in PreG, and 80.2% in SynG; however, these differences were not significant *p* > 0.05.

### 3.3. Histological Examination of Rumen

We observed significant differences between papillae length and width, the thickness of the ruminal epithelium, and the thickness of stratum corneum of rumen saccus dorsalis as well as between the length of papillae, the thickness of the ruminal epithelium, and the thickness of stratum corneum of rumen saccus ventralis (Table 3).

The mucosa of the abomasum in the cardia zone was significantly thicker in the synbiotic group and prebiotic group than in the control group. In the CoG group, the thickness of abomasum in the cardia zone was Me = 510 (IQR 472–558) *μ*m, Me = 571 (IQR 571) *μ*m in PreG, and Me = 610 (IQR 585–642) µm in the SynG group, *p* < 0.001, but the thickness of mucosa in fundus zone in SynG Me = 673 (IQR 632–752) µm was significantly thicker than in CoG Me = 581 (IQR 515–657) *μ*m and in PreG Me = 620 (IQR 575–660) *μ*m, *p* < 0.001. The thickness of mucosa of abomasum in the pyloric gland zone in PreG Me = 912 (IQR 852–1100) *μ*m was significantly thicker than in both CoG Me = 834 (IQR 787–896) and SynG Me = 854 (IQR 792–900), *p* < 0.001.

### 3.4. Histological Examination of Small and Large Intestine

There were significant differences between all treatment groups in such parameters as the thickness of the mucosa of the middle part of the duodenum, the end of the jejunum, and the thickness of the whole wall in the end of the jejunum. The other parameters significantly differed between the groups (Table 4).

## 4. Discussion

Prebiotic inulin and our novel synbiotic (prebiotic inulin and yeast *Saccharomyces cerevisiae* strain 1026) had a significant impact on pH in different parts of the gastrointestinal canal in calves and significantly improved the development of its different microscopic structures but did not affect the weight and relative weight of different parts of the stomach.

Prebiotics and probiotics can impact the pH in the whole gastrointestinal canal. Krol [24] tested a prebiotic mannan oligosaccharide (MOS) at the dose of 4 g/day/head and documented that it significantly increased rumen pH compared to the group of calves which did not receive this prebiotic. He also tested prebiotic inulin in amounts of 3 and 6 g/day/head and documented similar results. Our results are in accordance with those of Krol [24]; we also documented higher rumen pH in calves which received prebiotic inulin compared to the control group.

Yeast *S. cerevisiae* uses oxygen creating a more anaerobic environment required by ruminal microorganisms and stimulates their growth, thereby indirectly stabilizing ruminal pH [25, 26]. Therefore, the yeast itself not only functions as a probiotic but also helps other rumen community members grow, and, thus, acts as a type of prebiotic. Yeast *S. cerevisiae* also reduces the availability of glucose which is needed for *S.bovis* to synthesize lactate and increases the utilization of l-lactate by *M. elsdenii* [27]. Ishaq et al. [28] fed active dry yeast to the dairy cows and recorded an increase in pH and rumen protozoal abundance. The increase in rumen pH could also be explained by partial defaunation of the rumen ecosystem as a result of brewer's yeast addition [29]. Specific yeast product *Yea-Sacc* 1026 increases the microbiologic activity of rumen via nitrogen metabolism changes in the rumen (reduction of ammonium concentration) [30], but hydrolyzed yeast stimulates proliferation of rumen bacteria [31, 32].

We have documented lower rumen pH in calves which received an additional supplement of synbiotic (inulin and *S. cerevisiae* strain 1026) to their diet when compared to the control group and the prebiotic group. These results are contrary to all study results described above, suggesting that yeast in combination with inulin does not increase rumen pH.

Probiotics not only positively impact rumen but also can enhance intestinal health. These additives stimulate the development of a healthy microbiota, prevent enteric pathogens from colonizing the intestine, increase digestive capacity, lower the pH, and improve mucosal immunity [33].

In the study reported by Tzortzis et al. [34], prebiotic inulin at the dose of 40 g/kg added to pigs' feed for 33–35 days caused a significant increase in *Bifidobacterium* count, increase in acetic acid level, and reduction in intestinal pH, compared to the control group. The observation was in agreement with the results reported by Júskiewicz et al. [35]. These authors observed the reduction in the intestinal pH in turkeys administered fructooligosaccharides (FOS) for 8 weeks at a concentration of 2%. These results are contrary to ours. We documented slightly higher pH in jejunum and colon compared to the control group, but we observed the reduction of pH in jejunum and colon in calves supplemented with synbiotic; the difference of pH in the colon in the synbiotic group significantly differed from the control group.

There is ambiguous information about the effect of *S. cerevisiae* yeast on the structural and functional development of the rumen. Supplementation of the yeast culture at 2% in a 42-day- long study slightly improves rumen development in calves [36]. In this experiment, the authors recorded an increase in papillae length and width at the time of weaning off, but this increase was not significant. Brewer et al. [37] in his 5-week long experiment found that *S. cerevisiae* fermentation products at the dosage of 1 g/head/day and 3.5 g/head/day enhance ruminal papilla length and width in calves. This observation was in agreement with the results reported by Xiao et al. [11] who performed 52-day long experiment by using *S. cerevisiae* fermentation products at the dosage of 0.5% and 1%. Besides, the authors documented the reduction of crypt depth of jejunum in calves supplemented with *S. cerevisiae* fermentation product.

Interesting results were observed in experiments with sheep. In examination with adding *S. cerevisiae* 2 g/kg dry matter (DM), MOS, 2 g/kg DM, and combination of this yeast and MOS (2 g/kg DM of *S. cerevisiae* + 2 g/kg DM of MOS) to sheep diet for 42 days revealed that the synbiotic treatment group had thinner *stratum corneum* in rumen compared to the control group. The total thickness of ruminal epithelium was lower in the group of sheep which received MOS than in a group of sheep which received *S. cerevisiae* additive. The thickness of *stratum corneum* was increased in the control group and in the *S. cerevisiae* group compared with the MOS and synbiotic groups, but these additives did not influence the widths of the papillae [38].

We documented a significant increase in the width of papillae in rumen *saccus dorsalis* and a significant increase in the length of papillae in rumen *saccus ventralis* in the synbiotic group compared to the control group. However, the prebiotic group showed better results in both the length of papillae and the width of papillae in rumen *saccus dorsalis*, but no significant differences in these parameters in rumen *saccus ventralis* when compared to the control group. We documented that the length of papillae in rumen *saccus dorsalis* and in rumen *saccus ventralis* was significantly greater in the synbiotic group than in the group which received just prebiotic inulin suggesting that the addition of yeast *S. cerevisiae* to inulin could potentially increase the rate of rumen papillae development. The thickness of the ruminal epithelium and *stratum corneum* in rumen *saccus dorsalis* and *ventralis* was significantly lower in calves of the control group compared to the group of calves receiving a combination of prebiotic inulin and probiotic *S. cerevisiae* strain 1026 and single prebiotic inulin. Our results are contrary to those of Magalhães et al. [39]; these authors observed no effect of yeast culture (2% of DM) on rumen development and function in a 70-day long experiment. Similar results are reported by Kaldmae et al. [40]. The authors observed no differences in calves' papillae length and width, rumen wall thickness at either one or two months of age when supplemented with *S. cerevisiae* (2% of DM) for 2 months.

There is very little information about the impact of *S. cerevisiae* yeast on hindgut digestion in ruminants. It could be because it is thought that yeast culture functions mainly in the rumen. In the study with lambs, Durand-Chaucheyras et al. [41] found that yeast cells remain alive in hindgut, suggesting that effects of yeast may be seen in different parts of the gastrointestinal canal, not just rumen. These results are in agreement with the results reported by Xiao et al. [11]. The authors found a significant increase in villus height of duodenum, jejunum, and ileum and a decrease in crypt depth of all parts of the small intestine in calves receiving *S. cerevisiae* fermentation products in doses 0.5% and 1% of DM for 52 days. Similar results were observed in research with piglets. The authors claimed that dietary supplementation of *S. cerevisiae* fermentation products (5 g/kg for 21 days) resulted in greater jejunal villus height compared with the control group in piglets [42]. *Saccharomyces cerevisiae* fermentation product (2.5 g/kg for 42 days) improved villus height in duodenum and jejunum, a crypt depth in duodenum, jejunum, and ileum in broilers [43]. These results are in agreement with those of our experiments. We noticed significant differences between synbiotic and control groups in such parameters as the thickness of the whole wall of the middle part of duodenum, jejunum, ileum, and colon and the thickness of mucosa (villi height and crypt depth) of the middle part of jejunum and crypt depth in the colon. All of these parameters were significantly greater in calves of the synbiotic group. Taller villi indicate more mature epithelium and enhanced absorptive function due to the increased absorptive area of the villus. Greater villus height increases the activities of enzymes secreted from the tips of the villi, resulting in improved digestibility [44].

There are several studies about the impact of prebiotic inulin on intestinal morphological changes. Masanetz et al. [45] reported that jejunum and ileum villus was shorter in 20-week long experiments with calves which received prebiotic 2% inulin, but villus in 2% lactulose treated animals was longer compared to the control group. These results are in agreement with those reported by Pierce et al. [46]. They reported a similar decrease of villus length after the addition of inulin (15 g/kg) to the feed of weaning piglets for 28 days. However, Rehman et al. [47] implied that inulin has a positive impact on intestine. These authors conducted a study with broiler chickens and found that 1% inulin in a 5-week long trial significantly increases the length of jejunum villi and results in deeper crypts in the inulin group. Our results are in agreement with Rehman et al. [47]. We recorded that the thickness of the whole wall (villi + crypts) in the middle part of duodenum, jejunum, and ileum as well as the thickness of mucosa in the middle part of jejunum and ileum was significantly greater in the prebiotic group than in the control group. The positive effect of prebiotics on small and large intestine mucosal growth and development could be explained by the synthesis of polyamines from prebiotics which are essential for intestinal development [48] or by the fermentation of inulin that provided short-chain fatty acids. These acids can also promote the proliferation of cells of the large and small intestine and, as a result, increase villus height and crypt depth [49].

## 5. Conclusion

We concluded that prebiotic inulin (6 g per head) and our novel synbiotic (6 g prebiotic inulin and 5 g yeast *Saccharomyces cerevisiae* strain 1026) impact pH in different parts of the gastrointestinal canal in calves. The addition of prebiotic inulin to calves' diet causes the increase of pH in rumen, abomasum, and intestines but when inulin was added to *S. cerevisiae*, pH decreased and was even lower than in the control group. Prebiotic inulin and its synbiotic with yeast *S. cerevisiae* positively impact the development of almost all morphological structures of rumen *saccus dorsalis*, rumen *saccus ventralis*, and intestine; moreover, calves from the synbiotic group showed better results in virtually all parameters. However, both inulin and synbiotic did not affect the weight and relative weight of different parts of the stomach. As the results of our study are very promising but the significance of these results was not always clear, we suggest continuing this research with an increased number of examined animals to confirm our findings.

## Figures and Tables

**Table 1:** The pH in different parts of the gastrointestinal canal.

Place of measurement	pH (mean ± SD)			*p* value ANOVA
	CoG	PreG	SynG	
Mouth cavity	7.9 ± 0.25^ac^	8.6 ± 0.35^b^	7.6 ± 0.21^c^	0.009
Rumen *atrium ruminis*	7.1 ± 0.12^ab^	7.5 ± 0.85^b^	4.7 ± 0.15^c^	0.001
Rumen ventral sac	7.1 ± 0.10^ab^	7.5 ± 0.60^b^	4.8 ± 0.30^c^	<0.001
Abomasum close to the pyloric sphincter	2.7 ± 1.07	3.6 ± 0.35	2.1 ± 0.51	0.092
Middle part of the abomasum	2.1 ± 0.25^ac^	3.8 ± 0.70^b^	2.0 ± 0.35^c^	0.007
Middle part of jejunum	6.3 ± 0.15	7.0 ± 0.70	6.0 ± 0.06	0.070
Middle part of colon	6.0 ± 0.12^ab^	6.5 ± 0.38^b^	5.3 ± 0.15^c^	0.003

^a−c^Means within a row without common superscripts are significantly different (*p* < 0.05). SD = standard deviation; CoG = control group; PreG = prebiotic group; SynG = synbiotic group.

**Table 2:** Weight (kg) and relative weight of different parts of the stomach (% of body weight).

Parameter (kg) (mean ± SD)	CoG	PreG	SynG	*p* value
Whole stomach	3.9 ± 0.53	4.4 ± 0.4	3.6 ± 0.22	0.525
Forestomachs	3.2 ± 0.39	3.7 ± 0.3	2.9 ± 0.38	0.830
Abomasum	0.6 ± 0.14	0.6 ± 0.1	0.7 ± 0.16	0.423

Parameter (% of body weight) (mean ± SD)				
Whole stomach	3.5 ± 1.17	3.4 ± 0.23	3.0 ± 0.37	0.505
Forestomachs	2.9 ± 0.93	2.9 ± 0.19	2.4 ± 0.47	0.669
Abomasum	0.6 ± 0.25	0.5 ± 0.04	0.6 ± 0.10	0.412

SD = standard deviation; CoG = control group; PreG = prebiotic group; SynG = synbiotic group.

**Table 3:** Results of histological examination of the rumen.

Localization	Parameters (*μ*m)	CoG		PreG		SynG		*p* value K–W test
		Median	Q1–Q3	Median	Q1–Q3	Median	Q1–Q3	
Rumen *saccus dorsalis*	The length of papillae	1016^ac^	765–1692	899^b^	668–1098	1033^c^	764–1525	0.004
	The width of papillae	282^a^	246–341	344^bc^	312–431	371^c^	310–475	<0.001
	The thickness of ruminal epithelium	77^a^	66–86	122^b^	101–171	153^c^	123–176	<0.001
	The thickness of stratum corneum	9^a^	7–12	17^b^	14–22	33^c^	24–38	<0.001

Rumen *saccus ventralis*	The length of papillae	1113^ab^	850–1578	1359^b^	831–2036	1647^c^	1304–2203	<0.001
	The width of papillae	334	275–395	346	270–413	322	287–377	0.861
	The thickness of ruminal epithelium	97^a^	72–127	129^bc^	99–177	120^c^	102–164	<0.001
	The thickness of stratum corneum	9^a^	7–12	25^bc^	15–32	29^c^	24–36	<0.001

^a−c^ Means within a row without common superscripts are significantly different (*p* < 0.05) Q1–Q3 = Quatile 1–Quartile 3; CoG = control group; PreG = prebiotic group; SynG = synbiotic group.

**Table 4:** Results of histological examination of intestines.

Localization	Parameters (*μ*m)	Group						*p* value K–W test
		CoG		PreG		SynG		
		Median	Q1–Q3	Median	Q1–Q3	Median	Q1–Q3	
The middle part of the duodenum	The thickness of mucosa (villi + crypts)	917	799–1066	929	819–1093	952	874–1048	0.628
	The thickness of the whole wall	1595^a^	1513–1665	1981^bc^	1800–2029	2042^c^	1922–2125	<0.001

The middle part of the jejunum	The thickness of mucosa (villi + crypts)	1647^a^	1157–2320	2234^bc^	1740–2455	234^6c^	1815–2635	<0.001
	The thickness of the whole wall	2412^a^	1931–2858	3154^bc^	2967–3550	3197^c^	2935–3579	<0.001

The end part of the jejunum	The thickness of mucosa (villi + crypts)	1216	1147–1414	1193	1079–1363	1211	1107–1326	0.641
	The thickness of the whole wall	1907	1758–4199	2162	1922–2728	2580	2258–3111	0.165

The middle part of the ileum	The thickness of mucosa (villi + crypts)	1632^a^	1559–1777	2983^bc^	2413–3581	2889^c^	1743–3259	<0.001
	The thickness of the whole wall	969^ac^	895–1023	1601^b^	1403–1855	1073^c^	953–1217	<0.001

The middle part of the colon	The crypt depth	612^ab^	566–671	659^b^	618–721	764^c^	729–799	<0.001
	The thickness of the whole wall	1371^ab^	1232–1481	1325^b^	1280–1423	1758^c^	1665–1935	<0.001

^a−c^Means within a row without common superscripts are significantly different (*p* < 0.05) Q1–Q3 = Quatile 1–Quartile 3; CoG = control group; PreG = prebiotic group; SynG = synbiotic group.

## Data Availability

The data used to support the findings of this study are available from the corresponding author upon request.
